# Decisions to decline breast screening and/or breast cancer treatment based on the potential harms of overdiagnosis and overtreatment: a qualitative study

**DOI:** 10.1136/bmjopen-2024-089155

**Published:** 2024-12-10

**Authors:** Shavez Jeffers, Alison Pilnick, Natalie Armstrong

**Affiliations:** 1 Diabetes Research Centre, University of Leicester, Leicester, UK; 2 School of Nursing and Public Health, Manchester Metropolitan University, Manchester, UK; 3 Department of Population Health Sciences, University of Leicester, Leicester, UK

**Keywords:** Decision Making, QUALITATIVE RESEARCH, Mass Screening, PUBLIC HEALTH

## Abstract

**Abstract:**

**Objectives:**

To explore the experiences of women who have made the decision to decline breast screening and/or breast cancer treatment for overdiagnosis/overtreatment reasons after being invited to the National Health Service Breast Screening Programme (NHS BSP).

**Design:**

Qualitative interview study using reflexive thematic analysis.

**Setting:**

Participants were recruited via social media, online forums and word of mouth. Semi-structured interviews were conducted between May 2021 and April 2022.

**Participants:**

20 women aged between 49 and 76 years old who had declined one or more of the following after receiving an invitation to participate in the NHS BSP: (1) screening investigation, that is, mammogram; (2) further investigations, for example, biopsy, ultrasound; (3) treatment, for example, mastectomy, chemotherapy, radiotherapy and (4) any other medical intervention, for example, ongoing medication.

**Results:**

The three main themes were as follows: (1) the perception that the NHS BSP information was biased towards uptake and so constrained choice; (2) taking an active role in decision-making by considering the benefits and harms of the NHS BSP and (3) navigating potential regret for having declined.

**Conclusions:**

In-depth understanding of the potential harms of overdiagnosis and overtreatment influenced the decision to decline for these participants and contributed to their dissatisfactions with the way that information was presented in the invitation to the NHS BSP and the women felt confident in their assessments of the benefits and harms. These findings differ from previous studies, which have suggested that the vast majority lack knowledge and understanding of what overdiagnosis and overtreatment are whereas the participants in this study demonstrated high levels of health literacy. Findings have implications for the way informed choice is considered and constructed in relation to the NHS BSP.

**STRENGTHS AND LIMITATIONS OF THIS STUDY**

- The study captured in-depth insights into the views of a population that chose to decline the NHS Breast Screening Programme and/or subsequent interventions, which are seldom heard in previous research.
- The number of participants was small; therefore, it cannot be stated that thematic saturation was achieved.
- The over-representation of participants with a healthcare/scientific background is very likely an expression of self-selection bias.

## Introduction

 The decision-making process behind actively declining or opting out of cancer screening programmes (as opposed to non-attendance for other reasons) is a complex interplay of personal values, knowledge and perceptions of potential benefits and harms.^1^,^3^ Within the UK, breast cancer screening is offered through the National Health Service Breast Screening Programme (NHS BSP), a population-based screening programme that invites eligible women from the age of 50 to their 71st birthday to participate every 3 years.^1^ The aim of the NHS BSP is to reduce mortality from breast cancer by diagnosing cancer at an early stage when treatment is more likely to be successful.^1^ The NHS BSP is offered by sending eligible women a postal invitation to attend routine screening; this can include either a preset appointment or an open invitation with instructions on how to book an appointment.^2^ The invitation letter is also required to include an information leaflet explaining what participation in the programme involves, information about the benefits and potential harms of having routine breast screening and explicitly stating that women should make an informed choice about whether to participate.^3^

Potential harms of the NHS BSP can include overdiagnosis and overtreatment. Overdiagnosis of breast cancer can be understood as the detection of a breast cancer at screening that would not have become clinically apparent in the person’s lifetime by usual care.^4^ An overdiagnosed case involves a tumour that fulfils the pathological criteria for a breast cancer and can only be identified if either the cancer never progresses (or regresses) or if the cancer progresses slowly enough that the individual dies of other causes before the cancer becomes symptomatic.^5^ For example, ductal carcinoma in situ (DCIS) is a precursor of invasive breast cancer,^6^ which means that while it may lead to invasive cancer, not all cases will progress.^7^ DCIS is often detected during mammograms as part of the NHS BSP^6^ as it is the earliest form of breast cancer, even though most women show no symptoms.^8^ Breast screening cannot identify overdiagnosed cases of breast cancer because progression of cancer tumours can vary.^5 9^ Overdiagnosis can lead to overtreatment, which can be defined as unnecessary treatment for a condition that is not life-threatening or would never cause any symptoms and may lead to harmful side effects^10^ such as physical harms from medical procedures,^11^ negative impact on well-being^12^ and reduced quality of life.^13^ Currently, predicting whether treatment can be avoided in a particular woman diagnosed with a breast cancer is not possible.^14^ Therefore, a concern is that detecting the same medically defined condition may benefit some if they are treated, while also harming others where the case would not have developed into symptoms or disease.^15^

Under the NHS BSP, women are faced not only with making an informed choice about whether to participate in the NHS BSP but also a series of choices following their initial decision: whether to undergo future screening investigations, treatment or other recommended medical interventions. Government guidelines have highlighted the importance of ensuring that women are provided with accessible information about the potential benefits and harms of the NHS BSP to enable them to make an informed choice.^16 17^ However, the information contained in invitations to screening has long been criticised for not being sufficiently balanced and thereby undermining informed choice, both in the UK and internationally.^18 19^ There is a recognised tension between informing women about the potential harms of overdiagnosis and overtreatment to facilitate informed choice and encouraging them to attend.^20 21^ Within the UK, it has been argued that the emphasis has been placed on encouragement to participate in the NHS BSP,^22^,^24^ which raises questions about the experiences of those who make a decision to decline screening, follow-up tests and/or breast cancer treatment.

Previous literature on cancer screening programmes that have examined non-attendance has used different terms for this group of people, including refusers, non-attenders, non-adherers, decliners and non-participants.^25^,^27^ Some studies have developed subcategories to distinguish between the varying reasons for individuals not attending or not accepting the medical intervention that is offered, for example, Marlow *et al*’s work^28^ with non-participants in cervical cancer screening where different groups were categorised as follows: unaware, unengaged, undecided, decliners and intenders. The labels given to these subcategories demonstrate not only the range of terminology but also that these terms can include a wide range of individuals and not just those who actively choose to decline. For example, individuals who forget to attend, those who are unsuitable for the screening programme (eg, those who have had a hysterectomy would be unsuitable for cervical cancer screening), those who had never heard of the screening programme,^29^ those who participate in screening but not in response to invitations or at the prescribed intervals (eg, self-referred and/or private healthcare)^30^ and those who have structural barriers preventing them from attending (eg, lack of transport) and so do not make their decision on health grounds.^31^

Not all research studies that focus on those who decline explicitly state their reason for selecting a specific term or describe the range of individuals who participated in their research. However, of the previous studies that have focused on non-attendance at breast cancer screening or breast cancer treatment, many have been conducted outside of the UK in countries such as the USA,^32^,^36^ Canada,^37^ Indonesia^38^ and Taiwan.^39^ Different countries offer different breast screening services and due to varying healthcare contexts, the breast cancer treatment options offered also vary. For example, the USA and Canada require individuals to have health insurance or to pay a fee for breast cancer services.^40 41^ Since this has been found to be an influencing factor of non-attendance at cancer services,^26^ it is difficult to determine whether the findings from those studies are applicable in a UK context.

Therefore, the aim of this study is to explore the experiences of women who have made the decision to decline breast screening and/or breast cancer treatment for overdiagnosis/overtreatment reasons after being invited to the NHS BSP.

## Methods

### Sampling and recruitment

A Standards for Reporting Qualitative Research checklist was used to guide the reporting of this qualitative study. 20 participants voluntarily agreed to take part in this study. Women were eligible to participate if they had declined one or more of the following after receiving an invitation to participate in the NHS BSP: (1) screening investigation ie, mammogram; (2) further test, for example, biopsy, ultrasound; (3) treatment for example, mastectomy, chemotherapy, radiotherapy and/or (4) any other medical intervention, for example, ongoing medication. Exclusion criteria were (1) those who were unable to speak or understand English, that is, anyone who would require an interpreter and/or (2) those who were terminally ill—this is because it can be deemed insensitive to include this population in a study of healthcare decision-making.^42^

Identifying and recruiting individuals who have declined screening and/or treatment for research is challenging due to the lack of a clear sampling frame. Initially, recruitment efforts involved sending direct messages to third-sector organisations and Facebook groups supporting women with breast cancer or menopause, as menopausal women are often within the age range invited to the NHS BSP.^3 43^ Additionally, study information was posted on forums such as Menopause Matters, Mumsnet and Netmums. However, identifying suitable participants proved difficult.

To address this, recruitment strategies were adjusted to include sharing the study details with an online discussion group for healthcare professionals interested in overdiagnosis and overtreatment. The study was also promoted on social media platforms, including Facebook and X (formerly Twitter). Information was further disseminated through word of mouth. Participants who expressed interest were sent detailed information, and all eligible volunteers were recruited after completing a consent form prior to data collection.

Data saturation is commonly used within qualitative research and typically refers to the process of sampling and analysing data until nothing new is generated.^44^ However, this can be problematic because it suggests completeness of understanding and a determinable fixed point for stopping data collection.^45^ Due to the recruitment challenges of accessing this particular population, as discussed above, and the fact that most/all of the participants had similar characteristics, it was not possible to recruit a more diverse population, so the decision to stop was based on the fact that those coming forward were more of the same.

### Data collection

Semi-structured interviews were conducted between May 2021 and April 2022 either online via Microsoft Teams (7) or over the phone (13), depending on participant preference (see online supplemental table 1). A topic guide was used to steer the interviews, in relation to the general order of the questions and the topics covered. The topic guide included questions about the experience of declining and the decision-making process—full details of the topic guide can be found in online supplemental files 1.

All the interviews were audio recorded via an encrypted digital recording device or online software. The audio recordings were transcribed verbatim,^46^ which involved the removal of identifiable information and allocating a pseudonym to each participant (as used in the Results reported here).

### Patient and public involvement

Prior to recruitment and data collection, an individual with lived experience and expertise in the area of declining due to potential overdiagnosis and/or overtreatment of breast cancer was approached to give feedback on the design of the study and also to give insights on the best way to recruit from the targeted population. In addition, prior to conducting the semi-structured interviews, a topic guide was created and used for a pilot interview with the same individual. The feedback from the pilot informed further refinements to the topic guide.

### Analysis

As the focus was to explore lived experiences from individual perspectives, reflexive thematic analysis was chosen as the most suitable method for data analysis.^47^ Analysis of the whole interview dataset involved coding supported by the software NVivo V.12, with codes generated inductively from topics raised by participants. Following this, key themes were developed then explored within the wider dataset to establish the veracity of these themes and identify deviant cases, with the themes subsequently refined. These themes were synthesised to understand shared views of declining breast screening and/or breast cancer treatment, aided by reference to existing published social science and health screening literature about participation in screening.

### Researchers’ description

Data collection and analysis were part of SJ’s PhD project, which was supervised by NA and AP. At the time of data collection and analysis, SJ had never been invited to the NHS BSP due to age and had no family history of breast cancer. The academic background of SJ before starting this research included health studies (BA Hons) health psychology (MSc) and social science research (MSc). No previous academic or employment experiences were focused on breast screening or breast cancer treatment. SJ had no prior relationship with study participants.

## Results

### Summary

All women who were interviewed had received invitations to the NHS BSP, but the point at which they declined to engage with the service differed. Some of the women declined all invitations to the NHS BSP from the outset, while others participated in the programme initially but then decided to decline future screening invitations. Other women accepted some or all screening invitations but decided to decline follow-up tests and/or treatment after having an anomaly identified. Overall, the women who participated in this study displayed an in-depth awareness of the potential benefits and harms of participating in the NHS BSP and the majority of the women had an understanding of the concepts of overdiagnosis and overtreatment. The harms of overdiagnosis and overtreatment were discussed as influencing factors in the decision to decline the invitation to the NHS BSP and/or breast cancer treatment, both explicitly through use of these terms and implicitly in the way they articulated the concerns that led to declining.

### The perception that the NHS BSP information was biased towards uptake, so constraining choice

All the women were asked ‘how do you feel about the way that the NHS BSP was offered to you?’ In response, some participants discussed how they felt as though there was a lack of recognition of choice and that the information with which they were provided assumed attendance to the programme. A perceived lack of information provided on the possible harms of overdiagnosis and overtreatment was also expressed. This framing contributed towards the perception that the information presented was biased, adding to some of the women’s displeasure about the way the invitation made them feel, as Jodie describes below:

Uhm, I, I was unhappy that the erm, that the information given is, is biased towards uptake. (Jodie-declined screening)

In the quote above, Jodie believed that the information she was given aimed to encourage women to accept the offer rather than providing neutral information to enable informed decision-making. When talking about the information and how they assessed it, women focused not only on what was included but also what they perceived had been omitted.

For some of the women, the perception that the information that they were provided with was biased made the decision-making process challenging, as the following quote from Christine illustrates:

I don’t think the information in the letter that they send out is fair. It’s really hard. It makes decision making much more difficult. (Christine-declined screening)

In the above quote, Christine did not explicitly disclose here what aspects of the information made it more difficult, however, when interviewed she did go on to discuss how she had access to other sources of information (such as evidence-based literature and having conversations with others) and how these helped her to make her decision.

Another aspect of the invitation that was perceived as biased towards uptake by the women was the preset appointment that some received. Preset appointments were commonly sent out with the invitation to the NHS BSP prior to the pause of the NHS BSP in March 2020 due to COVID-19 restrictions. The purpose of sending a preset appointment was to encourage women to attend as research revealed that it was a suitable method to achieve higher rates of participation.^48^,^50^ However, some of the women who discussed receiving a preset appointment viewed it as another technique used to take away any notion of choice, for example, Sylvester below:

When you get your invite to go for screening you get an appointment and a time, it’s not like you make the decision. (Sylvester-declined treatment)

In the quote above, Sylvester reflected on the information that was included in her invitation and discussed how she did not feel as though she was being presented with a choice, as an appointment had already been booked for her. Even though formally this appointment can be declined, the fact it was already scheduled appeared to be interpreted by some as undermining the principle that they would make a choice.

### Taking an active role in decision-making by considering the benefits and harms of participating in the NHS BSP

The actions that the women described taking in order to make a decision emphasised their agency. Demonstrating an understanding of health information was discussed by some of the women, alongside accounts of how they had put thought into their decision by explaining that they asked questions in consultations with healthcare professionals, read additional and appropriate information and weighed up the benefits and harms based on that. This agency is demonstrated by Kathy below:

I’ve always been slightly, erm, independent minded shall we say, yeah… I’ll ask questions and I’ll think about it for myself and think about the pros and cons for me personally. (Kathy-declined screening)

In the quote above, Kathy presents herself as inquisitive and weighing up the benefits and harms in relation to her personal situation. Kathy presents her decision as a well-thought-out and conscious decision. Explanations of personal attributes that contributed to the decision-making process were also offered by other women, such as Fern:

I am an informed consumer, I read the papers… always ask a lot of questions and I come from a er, you know I’m, I’m very highly scientifically trained, I’m not a naïve observer in that sense. (Fern-declined screening)

Fern highlights that she read papers, which may be referring to research papers due to her discussing scientific evidence and health statistics whenever she mentioned papers throughout her interview. In stating this, Fern locates her decision as based on scientific knowledge and not a lack of understanding.

In addition, some of the women also discussed their knowledge of some of the methodological limitations of the research underpinning the effectiveness of the NHS BSP and how that was also considered in their decision-making process:

All the screening interventions are at risk of overdiagnosis. And it’s often a combination of lead time bias. Lead time bias is a really major factor because you know someone can always stand up and say people survive longer if they go for screening. That’s true, that’s just because they get their diagnosis earlier. It doesn’t mean that they’re in any way doing better, if you see what I mean. Yeah, I mean I know a lot about this. (Natasha-declined screening)

In the quote above, Natasha’s use of specific terminology demonstrates her awareness and understanding of the research methods used to determine the benefits and harms of the NHS BSP. Portraying agency and autonomy was evident throughout the women’s explanations and was multilayered, encompassing their need to do their own research before deciding whether to accept or not.

### Navigating potential regret for having declined

Two of the women (Laura and Sylvester) who were interviewed had gone on to develop invasive breast cancer. Both had chosen to accept their invitation for screening within the NHS BSP, had had further tests and been diagnosed with DCIS. Both had then made the decision to decline a mastectomy, requesting to have active monitoring instead. In both cases, 8–9 years after declining a mastectomy, they were diagnosed with an invasive breast cancer and subsequently accepted treatment. When asked whether they felt differently about their decision to decline a mastectomy now than they did when they initially made the decision, they made sense of how they felt about it in relation to how lucky (or unlucky) they had been. These women did not regret their decision to decline as they still felt that at the time of their diagnosis, they had made the right choice for them:

I think I’ve been unlucky in a lot of ways, but I’ve enjoyed, good healthy lifestyle on the whole through the, 8 or 9 years that I’ve had breast cancer. (Laura-declined treatment)

Laura described her eventual development of an invasive breast cancer in terms of being something outside of her control, that is, due to bad luck. Which was a perception that was also expressed by Sylvester:

And ‘cause you hope that you will be one of the lucky ones and that it doesn’t turn invasive erm, but I think my luck ran out. (Sylvester-declined treatment)

Both Laura and Sylvester did not regret their original decision to decline because they felt as though they were able to preserve their quality of life by avoiding side effects and harms of breast cancer treatment for several years, as explained further by Laura:

Everyone talks about fighting cancer, but my approach has very much been to live with cancer for as long as I can and accept that, eventually it probably will get me and I think I’ve had many years of, erm, and now it’s got me. Whereas, with the treatment, I think I would have had a much worse, less healthy life during the intervening period. (Laura-declined treatment)

The quote above demonstrates the way in which some women weighed up advantages and disadvantages even where invasive cancer was eventually diagnosed. Quality of life was important to Laura, and she believed that she had avoided the negative effects and potential harms of treatment for up to 9 years. Even though it is unclear whether Laura and Sylvester’s DCIS had been overdiagnosed as they could have potentially chosen to decline treatment that they needed, the quotes above demonstrate how their perception of overtreatment validated their decisions to decline.

## Discussion

### Principal findings

Overall, the women who participated in this study presented accounts of feeling that the NHS BSP invitation and the information that it included were not satisfactory due to the perceived emphasis on benefits, the limited information on harms and the persuasive framing of the information. They described how they felt the invitation was offered in a way that undermined the explicit presentation of choice because of the way benefits and harms were explained and, in some cases, the inclusion of a preset appointment. In this context, women presented ways in which they had demonstrated their agency, including seeking additional relevant information and exhibiting their familiarity with health-related information. Sophisticated understandings of the potential harms of overdiagnosis and overtreatment were presented as influencing factors on the decision to decline.

### Strengths and limitations

A strength of this study was the use of qualitative interviews that allowed for in-depth exploration of women’s accounts of declining cancer screening and/or treatment. The study recruited a population that has been missing from previous literature and perceived as a marginalised group.^51^ However, it also comprised an unusual sample as the majority had health-related occupations. Recruiting women with health-related occupations was not intentional. There were recruitment challenges in accessing this particular population, which resulted in the final sample having similar characteristics. The over-representation of participants with a healthcare/scientific background is very likely an expression of self-selection bias. An explanation for this could be that it is possible that women who make the decision to decline due to the potential harms of overdiagnosis and overtreatment are more likely to be those who are medically/scientifically trained because they have the skills and resources to understand and assess the research and literature exploring those harms. As this was a small sample, it cannot be stated that thematic saturation was achieved.

### Comparison with existing literature

This study is novel, as there are no studies that have specifically focused on exploring the experiences of women who have declined breast screening and/or breast cancer treatment after being invited to the NHS BSP. While there are studies in other countries that have focused separately on declining breast screening^32 52^ and declining breast cancer treatment,^34^,^3953^ it is difficult to compare findings because of the different healthcare systems involved, in terms of access, frequency and cost to the individual.^40 41^

A randomised controlled trial found that better information improves the chances that women invited to breast screening make choices that are in alignment with their values and preferences.^54^ This may explain how, for the women in this study, the awareness of the potential harms of overdiagnosis and overtreatment influenced the decision to decline. Previous studies examining non-attendance in breast cancer screening have found that women with lower levels of education are less likely to consider the harm of overdiagnosis.^55^ In addition, previous literature has also suggested that the organisation of BSPs may constrain choice, and therefore, encourage women to passively attend screening.^56^ However, the women in the present study had high levels of education, which suggests that they had higher health literacy than the general population. Therefore, it is possible that the vast majority lack knowledge of overdiagnosis but the subsample in the present study did not.

### Implications for practice and research

The women involved in this study may not be typical of the wider population invited to the NHS BSP, yet they illustrate the limitations of an informed choice model in the context of a programme that is reliant on high uptake for effectiveness. Overall, these women demonstrated how people can have sophisticated understandings of overdiagnosis and overtreatment.

## supplementary material

10.1136/bmjopen-2024-089155 online supplemental file 1

10.1136/bmjopen-2024-089155 online supplemental file 2

## Data Availability

No data are available.

## References

[1] Office for Health Improvement & Disparities (2021). Breast screening pathway requirements specification.

[2] Digital NHS (2022). NHS Breast Screening Programme, England 2020-21: COVID-19 impact during 2020-21.

[3] NHS England and NHS Improvement (2019). NHS public health functions agreement 2019-20: Service specification No. 24. NHS Breast Screening Programme.

[4] Monticciolo DL, Helvie MA, Hendrick RE (2018). Current Issues in the Overdiagnosis and Overtreatment of Breast Cancer. Am J Roentgenol.

[5] Welch HG, Black WC (2010). Overdiagnosis in Cancer. JNCI J Natl Cancer Inst.

[6] Cancer Research (2018). Ductal carcinoma in situ (DCIS). https://wwwcancerresearchukorg/about-cancer/breast-cancer/stages-types-grades/types/ductal-carcinoma-in-situ-dcis.

[7] Han MS, Khan SA (2018). Clinical Trials for Ductal Carcinoma In Situ of the Breast. J Mammary Gland Biol Neoplasia.

[8] Macmillan (2016). What is DCIS? - Understanding - Macmillan Cancer Support.

[9] Elmore JG, Fletcher SW (2012). Overdiagnosis in Breast Cancer Screening: Time to Tackle an Underappreciated Harm. Ann Intern Med.

[10] National Cancer Institute (2011). Definition of overtreatment - NCI Dictionary of Cancer Terms - NCI.

[11] Welch HG, Schwartz L, Woloshin S (2011). Overdiagnosed: making people sick in the pursuit of health.

[12] Davies L, Hendrickson CD, Hanson GS (2017). Experience of US Patients Who Self-identify as Having an Overdiagnosed Thyroid Cancer. *JAMA Otolaryngol Head Neck Surg*.

[13] Pickles K, Hersch J, Nickel B (2022). Effects of awareness of breast cancer overdiagnosis among women with screen-detected or incidentally found breast cancer: a qualitative interview study. BMJ Open.

[14] Helvie MA (2019). Perspectives on the Overdiagnosis of Breast Cancer Associated with Mammographic Screening. *J Breast Imaging*.

[15] Hofmann B (2016). Medicalization and overdiagnosis: different but alike. *Med Health Care and Philos*.

[16] Public Health England (2019). NHS population screening explained.

[17] Department of Health, Public health functions to be exercised by NHS England (2013). Service specification no.24. Breast screening programme.

[18] Gøtzsche PC, Hartling OJ, Nielsen M (2009). Breast screening: the facts--or maybe not. BMJ.

[19] Jørgensen KJ, Gøtzsche PC (2006). Content of invitations for publicly funded screening mammography. BMJ.

[20] Gigerenzer G (2015). Towards a paradigm shift in cancer screening: informed citizens instead of greater participation. *BMJ*.

[21] Waller J, Osborne K, Wardle J (2016). Enthusiasm for cancer screening in Great Britain: a general population survey. Br J Cancer.

[22] Edward Stefanek M (2011). Uninformed Compliance or Informed Choice? A Needed Shift in Our Approach to Cancer Screening. JNCI J Natl Cancer Inst.

[23] Gøtzsche PC, Jørgensen KJ (2011). The Breast Screening Programme and misinforming the public. J R Soc Med.

[24] Public Health England (2021). NHS breast screening Helping you decide.

[25] Artama M, Heinävaara S, Sarkeala T (2016). Determinants of non-participation in a mass screening program for colorectal cancer in Finland. Acta Oncol.

[26] Knight TG, Deal AM, Dusetzina SB (2018). Financial Toxicity in Adults With Cancer: Adverse Outcomes and Noncompliance. J Oncol Pract.

[27] Puts MTE, Tapscott B, Fitch M (2015). A systematic review of factors influencing older adults’ decision to accept or decline cancer treatment. Cancer Treat Rev.

[28] Marlow LAV, Chorley AJ, Rockliffe L (2018). Decision‐making about cervical screening in a heterogeneous sample of nonparticipants: A qualitative interview study. Psychooncology.

[29] Andreassen T, Weiderpass E, Nicula F (2017). Controversies about cervical cancer screening: A qualitative study of Roma women’s (non)participation in cervical cancer screening in Romania. Soc Sci Med.

[30] Worthley DL, Cole SR, Esterman A (2006). Screening for colorectal cancer by faecal occult blood test: why people choose to refuse. Intern Med J.

[31] Lang RS, Isaacson JH (1999). Screening. Med Clin N Am.

[32] Watson-Johnson LC, DeGroff A, Steele CB (2011). Mammography Adherence: A Qualitative Study. J Womens Health (Larchmt).

[33] Seaman K, Dzidic P, Breen L (2018). Exploring breast health practices of post-menopausal women: Implications to informed consent. J Health Psychol.

[34] Kim E, Jang SH, Andersen MR (2021). “I Made All Decisions Myself”: Breast Cancer Treatment Decision-Making by Receivers and Decliners. Asia Pac J Oncol Nurs.

[35] Weinmann S, Taplin SH, Gilbert J (2005). Characteristics of Women Refusing Follow-up for Tests or Symptoms Suggestive of Breast Cancer. J Natl Cancer Inst Monographs.

[36] Citrin DL, Bloom DL, Grutsch JF (2012). Beliefs and perceptions of women with newly diagnosed breast cancer who refused conventional treatment in favor of alternative therapies. Oncologist.

[37] Joseph K, Vrouwe S, Kamruzzaman A (2012). Outcome analysis of breast cancer patients who declined evidence-based treatment. World J Surg Oncol.

[38] Iskandarsyah A, de Klerk C, Suardi DR (2014). Psychosocial and cultural reasons for delay in seeking help and nonadherence to treatment in Indonesian women with breast cancer: a qualitative study. Health Psychol.

[39] Chen SJ, Kung P-T, Huang KH (2015). Characteristics of the Delayed or Refusal Therapy in Breast Cancer Patients: A Longitudinal Population-Based Study in Taiwan. PLoS One.

[40] Cancer Care Ontario (2024). Ontario Breast Screening Program (OBSP) - Cancer Care Ontario.

[41] Centers for Disease Control and Prevention (2023). What Is Breast Cancer Screening.

[42] Ritchie J, Lewis J (2003). Qualitative research practice: a guide for social science students and researchers.

[43] NHS (2017). Menopause.

[44] Green J (2014). Qualitative methods for health research.

[45] Braun V, Clarke V, Hayfield N (2019). Thematic analysis.

[46] Atkinson JM, Heritage J (1984). Structures of social action: studies in conversation analysis.

[47] Braun V, Clarke V (2014). What can “thematic analysis” offer health and wellbeing researchers?. Int J Qual Stud Health Well-being.

[48] Segnan N, Senore C, Giordano L (1998). Promoting participation in a population screening program for breast and cervical cancer: a randomized trial of different invitation strategies. *Tumori*.

[49] Allgood PC, Maroni R, Hudson S (2017). Effect of second timed appointments for non-attenders of breast cancer screening in England: a randomised controlled trial. Lancet Oncol.

[50] Duffy SW, Seedat F, Kearins O (2022). The projected impact of the COVID-19 lockdown on breast cancer deaths in England due to the cessation of population screening: a national estimation. Br J Cancer.

[51] Lagerlund M, Widmark C, Lambe M (2001). Rationales for attending or not attending mammography screening--a focus group study among women in Sweden. Eur J Cancer Prev.

[52] Seaman K, Dzidic PL, Castell E (2021). Subject positions in screening mammography and implications for informed choice. Psychol Health.

[53] Restrepo DJ, Sisti A, Boczar D (2019). Characteristics of Breast Cancer Patients Who Refuse Surgery. Anticancer Res.

[54] Hersch J, Barratt A, Jansen J (2015). Use of a decision aid including information on overdetection to support informed choice about breast cancer screening: a randomised controlled trial. Lancet.

[55] Gong J, Kampadellis G, Kong Q (2023). Factors determining non-attendance in breast cancer screening among women in the Netherlands: a national study. Health Promot Int.

[56] Long H, Brooks JM, Harvie M (2019). How do women experience a false-positive test result from breast screening? A systematic review and thematic synthesis of qualitative studies. Br J Cancer.

