# Supplementary material for: Decisions to decline breast screening and/or breast cancer treatment based on the potential harms of overdiagnosis and overtreatment: a qualitative study
Source: BMJ Open. 2024 Dec 10;14(12):e089155. doi: 10.1136/bmjopen-2024-089155 (PMC11647357; doi:10.1136/bmjopen-2024-089155)
Supplement: online supplemental file 1 [file bmjopen-14-12-s001.pdf]

Navigating perceived overdiagnosis and overtreatment: a qualitative study - Topic guide

Welcome and introduction:

Hello, my name is Shavez and I am a PhD student from the University of Leicester, can I confirm that I am speaking with ________.

Before we begin with the interview there are a couple of things that I would like to confirm with you?

Can you confirm that you have read the information sheet and the email consent statements.

Do you have any questions regarding the information that were emailed over to you?

Do you give your consent to those statement?

Are you happy with this interview being audio-recorded?

No personal identifiable data will be recorded and a participant number will be allocated to you and sent you through email after the interview.

Opening questions:

Tell me about your experience with breast cancer services?

Can you tell me about your experience with the NHS breast cancer screening programme up until now?

Can you tell me what intervention you declined (screening/treatment/follow up tests/other?)

The following questions refer to the intervention that you declined;

Could you tell me about how you were offered the intervention?

How long ago has it been?

How do you feel about the way that it was offered to you?

How did you make your decision to decline? One-off decision or definite decision?

Did you feel any pressure from anyone?

What sources of information guided your decision? Did you talk to anyone about it?

How do you feel about your decision now?

Do you feel differently now compared to when you first made the decision? What are the differences/similarities?

Did you tell anyone about your decision? Who did you tell? How did they respond? How did that make you feel?

Can you describe to me a conversation that you have had with a healthcare professional about your decision? Have you been prompted by healthcare professionals?

Since your decision, has your experience of interacting with healthcare professional changed?

Have you declined screening/treatment for breast cancer or any other service since then? Was that the first time you declined anything?

Have you considered or taken up any other services or avenues relating to breast cancer screening/treatment outside of NHS? For example, any alternative therapies.

Have you come across the idea of overdiagnosis? Could you explain what you know about it?

Anything not covered? Is there anything that we haven't covered in the interview that you think we should know or think about?

Closing and thanks – Are you still happy for you to use all the information provided?

You will receive an email, which will include your participant number and information about how to withdraw your contribution from the study.

Pseudonym? Own name?

Would you like to receive a summary of the findings? I will need to keep your contact details for this purpose

Is there anyone you know that might be interested in participating in this study?

Do you have any suggestions on how I could find more participants?

Thank you for your time and contribution.
