# Supplementary material for: Decisions to decline breast screening and/or breast cancer treatment based on the potential harms of overdiagnosis and overtreatment: a qualitative study
Source: BMJ Open. 2024 Dec 10;14(12):e089155. doi: 10.1136/bmjopen-2024-089155 (PMC11647357; doi:10.1136/bmjopen-2024-089155)
Supplement: online supplemental file 2 [file bmjopen-14-12-s002.docx]

# Supplementary 2: Table 1. - Characteristics of participants

| **Pseudonym** | **Age** | **Recruitment avenue** | **What was accepted/declined along the NHS BSP pathway in chronological order** | **Time since last declined** | **Reason for declining** | **Health-related occupation (self-described)** |
| --- | --- | --- | --- | --- | --- | --- |
| Laura | Late 70s | Word of mouth | Accepted screening invitations, diagnosed with DCIS*** via NHS BSP, declined mastectomy then accepted active monitoring/After 8-9 years diagnosed with BC**, accepted mastectomy | 9 years | Chose active monitoring instead due to lack of symptoms | Not known |
| Anna | Mid 50s | Overdiagnosis group | Accepted screening invitations then decided to decline future screening invitations | 3 years | Possibility of overdiagnosis and/or overtreatment | Geriatrician |
| Jodie | Late 50s | Twitter | Declined all screening invitations | 5 months | Influenced by medical professionals | Medical writer/editor |
| Kathy | Early 50s | Word of mouth | Declined all screening invitations | 2 years | Being at a low risk | No |
| Fern | Mid 60s | Twitter | Accepted screening invitations, diagnosed with BC** in interval between screenings, accepted mastectomy, chemotherapy, radiotherapy then declined adjunctive therapy & future screening invitations | 8 years | Possibility of overdiagnosis and/or overtreatment | General Practitioner (GP) |
| Shannon | Late 50s | Overdiagnosis group | Declined all screening invitations | 3-4 years | Being at a low risk/Possibility of overdiagnosis and/or overtreatment | General Practitioner (GP) |
| Donna | Late 50s | Twitter | Declined all screening invitations | 8 years | Potential harms outweigh the benefits | General Practitioner (GP) |
| Hannah | Late 60s | Word of mouth | Accepted screening invitations then decided to decline future screening invitations | 2-3 years | Possibility of overdiagnosis and/or overtreatment | Author on women’s health |
| Sam | Early60s | Overdiagnosis group | Declined all screening invitations | 12 years | Potential harms outweigh the benefits | Academic in women’s health |
| Joanna | Early60s | Word of mouth | Accepted screening invitations, diagnosed with BC via NHS BSP, accepted lumpectomy, mastectomy, biopsy then declined future screening invitations | 3 years | Possibility of overdiagnosis and/or overtreatment | Not known |
| Sylvester | Late 50s | Overdiagnosis group | Diagnosed with DCIS*** via NHS BSP, declined mastectomy then accepted active monitoring/After 9 years diagnosed with BC**, accepted mastectomy, chemotherapy, radiotherapy | 9 years | Possibility of overdiagnosis and/or overtreatment | Not known |
| Julia | Early 60s | Overdiagnosis group | Declined all screening invitations | 7 years | Possibility of overdiagnosis and/or overtreatment | Not known |
| Jess | Early 50s | Overdiagnosis group | Declined all screening invitations | 6 weeks | Possibility of overdiagnosis and/or overtreatment | General Practitioner (GP) |
| Wendy | Late 40s | Overdiagnosis group | Declined all screening invitations before & after identified with BRCA gene | 2 years | Possibility of overdiagnosis and/or overtreatment | General Practitioner (GP) |
| Erica | * | Overdiagnosis group | Accepted screening invitations then decided to decline future screening invitations | * | Possibility of overdiagnosis and/or overtreatment | General Practitioner (GP) |
| Natasha | Early 70s | Overdiagnosis group | Declined all screening invitations | A few months | Possibility of overdiagnosis and/or overtreatment | General Practitioner (GP) |
| Bridget | Early 60s | Breast Cancer charity forum | Accepted screening invitations, diagnosed with BC** via NHS BSP, radiotherapy, lumpectomy, drug therapy then declined drug therapy due to side effects | 6 months | Side effects of medication | Patient Advocate Volunteer at breast cancer charity |
| Christine | * | Twitter | Identified with dense breasts via NHS BSP (pilot trial) then declined future screening invitations | A few months | Potential harms outweigh the benefits | Health Journalist |
| Tracey | * | Twitter | Declined all screening invitations | 6 months | Possibility of overdiagnosis and/or overtreatment | General Practitioner (GP) |
| Emma | Early 50s | Word of mouth | Declined all screening invitations | 6 months | Potential harms outweigh the benefits | Not known |

*- = missing data

**BC=Breast Cancer

***DCIS=Ductal Carcinoma In Situ
