# Supplementary material for: Decisions to decline breast screening and/or breast cancer treatment based on the potential harms of overdiagnosis and overtreatment: a qualitative study
Source: BMJ Open. 2024 Dec 10;14(12):e089155. doi: 10.1136/bmjopen-2024-089155 (PMC11647357; doi:10.1136/bmjopen-2024-089155)
Supplement: Supplementary file 3 [file bmjopen-2024-089155.reviewer_comments.pdf]

PEER REVIEW HISTORY

BMJ Open publishes all reviews undertaken for accepted manuscripts. Reviewers are asked to complete a checklist review form and are provided with free text boxes to elaborate on their assessment. These free text comments are reproduced below.

ARTICLE DETAILS

Title (Provisional)

Decisions to decline breast screening and/or breast cancer treatment based on the potential harms of overdiagnosis and overtreatment: a qualitative study

Authors

Jeffers, Shavez; Pilnick, Alison; Armstrong, Natalie

VERSION 1 - REVIEW

Reviewer 1

Name Wang, Wei

Affiliation Food and Drug Administration, Division of Biostatistics, Center for Devices and Radiological Health

Date 29-Aug-2024

COI I have no completing interests

In this study, the authors included 20 women aged between 49-76 years old who had declined one or more invitations to participate in the NHS BSP in order to understand the overdiagnosis and overtreatment on breast screening and/or breast cancer treatment decisions. I do not agree that the conclusion provided in this manuscript is helpful and informative to BMJ Open readers due to the following reasons, (1) the sample size in this study is very small and only 20 women were enrolled in this study; (2) the rejection of invitations is related to four different BSP categories, screening investigations, further investigations, treatment, and any other medical invention; (3) the population that is comprised of a sample of participants who largely had health-related occupations is a biased population and the conclusion cannot generalize to a general target population; (4) the major conclusions and results made in the whole manuscript are from personal opinions of few participants. Although these several limitations were listed and mentioned by the authors, with limited useful information provided in this manuscript from a biased limited heterogenous small population, I do not agree that this manuscript will help understand the overdiagnosis and overtreatment on breast screening and/or breast cancer treatment decisions.

**Reviewer**	**2**
**Name**	**Jørgensen, Karsten Juhl**
**Affiliation**	**Rigshospitalet, The Nordic Cochrane Centre**
**Date**	**02-Sep-2024**
**COI**	**I confirma that I have no conflicts of interest**

bmjopen-2024-089155

This manuscript describes a qualitative study. It explores the reasoning behind the decision not to accept the invitation for the NHS Breast Screening Programme (NHS BSP); follow-up investigations due to NHS BSP results; or treatment suggested due to NHS BSP results.

The authors note that an over-representation of individuals with health care or scientific training among the respondents. This is probably not that surprising as it would likely take some level of professional confidence in the personal decision not to attend, given that the invitation, with a pre-specified appointment, conveys an expectation to attend and as most who receive i will likely think that someone who knows more than themselves have looked at the science and decided to offer the programme, a decision that in itself can be seen as a recommendation from a health authority, unless invitations specifically state that there is a real choice to be made. The narrative that ’screening catches it early and catching it early is good’ is very tough to counter as an individual.

The authors note that their study is novel and has a different focus. This is true, to the best of my knowledge. Contrary, I have seen countless studies that explore ’barriers to participation’, which, of course, implicitly implies that such barriers should be overcome so that more are screened and official goals for participation levels are met. Parts of the science too, and not only the invitations, have been biased in favour of participation.

Overall, the manuscript is well-written, though the formatting would benefit from more work.

Suggestions for changes:

Some sections are quite long and there are repetitions that do not seem necessary. Specifically, the Introduction section is quite wordy and could be shortened. Parts of the summaries of what the interviewees are saying simply repeat what they say. I find this unnecessary and think it would be more valuable to readers to cut the repetitions and perhaps find room for one or two more examples.

The Abstract concludes that ‘These findings differ from previous studies which have suggested that there is a lack of knowledge and understanding of what overdiagnosis and overtreatment is in relation to the NHS BSP.’ This may give the impression that one finding is correct and the other is lacking. However, given the type of participants you included, it is of

course perfectly possible that the vast majority lack knowledge of overdiagnosis but that your subsample did not. I am sure you are aware of this, but a clarification might be in place.

Since you had 20 participants, an overview of their reasons to decline participation could be summarized in a Table. As the manuscript stands now, the reader misses to see why participants that are not used as examples in the Results section made the choices they did.

The over-representation of participants with a healthcare/scientific background is very likely an expression of self-selection bias and this term could be used to describe this limitation in the relevant section of the Discussion.

Information in invitations to screening and official web sites, including in the UK, have been critized for a long time (20 years), including the pre-specified time of appointment and its implications for informed choice. Some tings have improved, including that overdiagnosis is now mentioned. This development could be acknowledged. Previous information is reviewed in these three studies:

Gøtzsche PC, Hartling OJ, Nielsen M, Brodersen J, Jørgensen KJ. Breast screening: the facts--or maybe not. BMJ. 2009 Jan 27;338:b86. doi: 10.1136/bmj.b86. PMID: 19174442.

Jørgensen KJ, Gøtzsche PC. Content of invitations for publicly funded screening mammography. BMJ. 2006 Mar 4;332(7540):538-41. doi: 10.1136/bmj.332.7540.538. PMID: 16513713; PMCID: PMC1388137.

Jørgensen KJ, Gøtzsche PC. Presentation on websites of possible benefits and harms from screening for breast cancer: cross sectional study. BMJ. 2004 Jan 17;328(7432):148. doi: 10.1136/bmj.328.7432.148. PMID: 14726344; PMCID: PMC314513.

These references are for your information and there is no expectation that you cite them. However, a very important RCT found that better information improves the chances that women invited to breast screening make choices that are in alignment with their values and preferences, and I do recommend that you cite this one:

Hersch J, Barratt A, Jansen J, Irwig L, McGeechan K, Jacklyn G, Thornton H, Dhillon H, Houssami N, McCaffery K. Use of a decision aid including information on overdetection to support informed choice about breast cancer screening: a randomised controlled trial. Lancet. 2015 Apr 25;385(9978):1642-52. doi: 10.1016/S0140-6736(15)60123-4. Epub 2015 Feb 18. Erratum in: Lancet. 2015 Apr 25;385(9978):1622. doi: 10.1016/S0140-6736(15)60304-X. PMID: 25701273.

Particularly as you informant in the planning phase, Hazel Thornton, is a co-author.

Page 11, Line 20-21: Given that they eventually developed invasive breast cancer, treating their screen-detected DCIS would not constitute overdiagnosis, by definition. Indeed, you define overtreatment in the Introduction as treatment that is unnecessary because the cancer was overdiagnosed. But their DCIS was very likely not overdiagnosed (unless their subsequently detected invasive breast cancer was overdiagnosed). As I read the statement

from the woman interviewed, she seems to be happy that her treatment, and thus associated harms, were postponed, granting her more years without them.

Reference 14 is authored not only by Gil Welch but equally by Lisa Schwartz and Steven Woloshin.

Karsten Juhl Jørgensen

**Reviewer**	**3**
**Name**	**Baines, Cornelia**
**Affiliation**	**University of Toronto Dalla Lana School of Public Health**
**Date**	**08-Sep-2024**
**COI**	**NONE.**

For four decades I have been a quantitative epidemiologist and truly am not acquainted with qualitative methodology. I accepted this review because of the subject.

Having reviewed innumerable manuscripts this is the first where I have not identified omissions, grammatic and spelling errors, or errors of fact. Quite wonderful.

Very impressive was the review of the literature.

The design was completely described.

The results were similarly complete, but for me resulted in a question:

*Twenty is not a huge population. When the presence of so many participants with medical credentials was noted, why weren't more people recruited?

VERSION 1 - AUTHOR RESPONSE

Reviewer	Comment	Response

Reviewer #1	In this study, the authors included 20 women aged between 49-76 years old who had declined one or more invitations to participate in the NHS BSP in order to understand the overdiagnosis and overtreatment on breast screening and/or breast cancer treatment decisions. I do not agree that the conclusion provided in this manuscript is helpful and informative to BMJ Open readers due to the following reasons, (1) the sample size in this study is very small and only 20 women were enrolled in this study; (2) the rejection of invitations is related to four different BSP categories, screening investigations, further investigations, treatment, and any other medical invention; (3) the population that is comprised of a sample of participants who largely had health-related occupations is a biased population and the conclusion cannot generalize to a general target population; (4) the major conclusions and results made in the whole manuscript are from personal opinions of few participants. Although these several limitations were listed and mentioned by the authors, with limited useful information provided in this manuscript from a biased limited heterogenous small population, I do not agree that this manuscript will help understand the overdiagnosis and overtreatment on breast screening and/or breast cancer treatment decisions.	Thank you for your feedback, but we respectfully disagree with the points you raise. We believe the conclusions provided are helpful and informative for the following reasons: 1) even though the sample size was small, it is not unusual for qualitative research and in-depth data was obtained which enabled new insights into this topic to be uncovered; 2) even though there are examples of rejection of four different BSP categories, the reasons for the decisions are often applicable across all stages of the pathway, as the participants were dissatisified with the screening programme as a whole and not just one element of it; 3) we acknowledge the participants recruited were a distinctive population, and declare this as a limitation, but we do not agree that means the analysis of their experiences is invalid; and 4) we believe this manuscript helps understand the concerns about overdiagnosis and overtreatment held by those who have chosen to decline and their experiences of making and enacting this decision.
Reviewer #2	Some sections are quite long and there are repetitions that do not seem necessary. Specifically, the Introduction section is quite wordy and could be shortened. Parts of the summaries of what the interviewees are saying simply repeat what they say. I find this unnecessary and think it would be more valuable to readers to cut the repetitions and perhaps find room for one or two more examples.	Thank you for this feedback. Areas of repetition from the Introduction and Results sections were removed, but adding text to respond to other points of feeback has meant there is not sufficient space to include additional examples.
Reviewer #2	The Abstract concludes that 'These findings differ from previous studies which have suggested that there is a lack of knowledge and understanding of what overdiagnosis and overtreatment is in relation to the NHS BSP.' This may give the impression that one finding is correct and the other is lacking. However, given the type of participants you	Conclusion section of abstract and comparison with existing literature section has been revised to provide clarification that the vast majority lack knowledge of overdiagnosis but my subsample did not

	included, it is of course perfectly possible that the vast majority lack knowledge of overdiagnosis but that your subsample did not. I am sure you are aware of this, but a clarification might be in place.	
Reviewer #2	Since you had 20 participants, an overview of their reasons to decline participation could be summarized in a Table. As the manuscript stands now, the reader misses to see why participants that are not used as examples in the Results section made the choices they did.	Thank you for the feedback. I have added an extra column in Table 1 titled 'Reasons for declining' and provided information for each participant
Reviewer #2	The over-representation of participants with a healthcare/scientific background is very likely an expression of self-selection bias and this term could be used to describe this limitation in the relevant section of the Discussion.	Thank you for this suggestion. A sentence has been added to the limitations section to state the possibility of self selection bias in the sample.
Reviewer #2	Information in invitations to screening and official web sites, including in the UK, have been critized for a long time (20 years), including the pre-specified time of appointment and its implications for informed choice. Some things have improved, including that overdiagnosis is now mentioned. This development could be acknowledged. Previous information is reviewed in these three studies: Gøtzsche PC, Hartling OJ, Nielsen M, Brodersen J, Jørgensen KJ. Breast screening: the facts--or maybe not. BMJ. 2009 Jan 27;338:b86. doi: 10.1136/bmj.b86. PMID: 19174442.<br>Jørgensen KJ, Gøtzsche PC. Content of invitations for publicly funded screening mammography. BMJ. 2006 Mar 4;332(7540):538-41. doi: 10.1136/bmj.332.7540.538. PMID: 16513713; PMCID: PMC1388137. Jørgensen KJ, Gøtzsche PC. Presentation on websites of possible benefits and harms from screening for breast cancer: cross sectional study. BMJ. 2004 Jan 17;328(7432):148. doi: 10.1136/bmj.328.7432.148. PMID: 14726344; PMCID: PMC314513. These references are for your information and there is no expectation that you cite them.	Thank you for this suggestion. A sentence has been added to the introduction to this effect, using some of these references.

Reviewer #2	However, a very important RCT found that better information improves the chances that women invited to breast screening make choices that are in alignment with their values and preferences, and I do recommend that you cite this one: Hersch J, Barratt A, Jansen J, Irwig L, McGeechan K, Jacklyn G, Thornton H, Dhillon H, Houssami N, McCaffery K. Use of a decision aid including information on overdetection to support informed choice about breast cancer screening: a randomised controlled trial. Lancet. 2015 Apr 25;385(9978):1642-52. doi: 10.1016/S0140-6736(15)60123-4. Epub 2015 Feb 18. Erratum in: Lancet. 2015 Apr 25;385(9978):1622. doi: 10.1016/S0140-6736(15)60304-X. PMID: 25701273.	Thank you for the suggestion. A sentence has been added to the Comparison with existing literature section using this reference.
Reviewer #2	Particularly as you informant in the planning phase, Hazel Thornton, is a co-author. Page 11, Line 20-21: Given that they eventually developed invasive breast cancer, treating their screen-detected DCIS would not constitute overdiagnosis, by definition. Indeed, you define overtreatment in the Introduction as treatment that is unnecessary because the cancer was overdiagnosed. But their DCIS was very likely not overdiagnosed (unless their subsequently detected invasive breast cancer was overdiagnosed). As I read the statement from the woman interviewed, she seems to be happy that her treatment, and thus associated harms, were postponed, granting her more years without them.	Thank you for the suggestion. Changes have been made to the final paragraph of the results section to clarify that even though it is possible that they declined treatment that was necessary, the perception of overtreatment validated the decision to decline in the quote.
Reviewer #2	Reference 14 is authored not only by Gil Welch but equally by Lisa Schwartz and Steven Woloshin.	Thank you. Changes made to the reference to include additional authors
Reviewer #3	Twenty is not a huge population. When the presence of so many participants with medical credentials was noted, why weren't more people recruited?	The first heading of the Methods sections has been changed to sampling and recruitment. Text has been added to this section that recognises this was a challenging group to identify and recruit, and to reflect on the size of the sample achieved.

VERSION 2 - REVIEW

**Reviewer**	**2**
**Name**	**Jørgensen, Karsten Juhl**
**Affiliation**	**Rigshospitalet, The Nordic Cochrane Centre**
**Date**	**19-Nov-2024**
**COI**	

The authors have addressed all my previous points in a satisfactory way and I recommend publication of the present manuscript.
